# Genetic Aspects of Congenital and Idiopathic Scoliosis

**DOI:** 10.6064/2012/152365

**Published:** 2012-12-31

**Authors:** Philip F. Giampietro

**Affiliations:** Waisman Center, University of Wisconsin-Madison, 1500 Highland Avenue, Madison, WI 53705, USA

## Abstract

Congenital and idiopathic scoliosis represent disabling conditions of the spine. While congenital scoliosis (CS) is caused by morphogenic abnormalities in vertebral development, the cause(s) for idiopathic scoliosis is (are) likely to be varied, representing alterations in skeletal growth, neuromuscular imbalances, disturbances involving communication between the brain and spine, and others. Both conditions are characterized by phenotypic and genetic heterogeneities, which contribute to the difficulties in understanding their genetic basis that investigators face. Despite the differences between these two conditions there is observational and experimental evidence supporting common genetic mechanisms. This paper focuses on the clinical features of both CS and IS and highlights genetic and environmental factors which contribute to their occurrence. It is anticipated that emerging genetic technologies and improvements in phenotypic stratification of both conditions will facilitate improved understanding of the genetic basis for these conditions and enable targeted prevention and treatment strategies.

## 1. Introduction

Advances in developmental biology have enabled improvements in the understanding of spine development and have provided contributions that enhance our understanding of genetic and environmental factors that are associated with congenital and idiopathic scoliosis. This paper will focus on salient features of both forms of scoliosis and highlight research focusing on genetic and environmental mechanisms for their occurrence.

## 2. Definitions and Epidemiology of Scoliosis

Idiopathic scoliosis (IS) is defined by the Scoliosis Research Society (http://www.srs.org/) as a lateral curvature of the spine of 10° or greater for which no cause can be determined. There is evidence that genetic and environmental factors are likely to play a role in the occurrence of both as described herin, although the mechanism responsible for this is uncertain at the present time. This rotational deformity is measured in the forward bending position by an inclinometer, and the scoliometer as an angle of trunk rotation (ATR). 

The incidence of idiopathic scoliosis (IS) in the general population ranges from 2% to 3%, varying with the definition of the magnitude of the curve. Population studies indicate that 11.1% of 1st degree relatives are affected, compared to 2.4% of 2nd degree, and 1.1% of 3rd degree relatives [1]. By age 16, 0.6% of affected people will have required active treatment with a full-time thoracolumbar-sacral orthosis (TLSO) or surgical correction with instrumentation [2, 3]. Older IS subclassification is based on the age of presentation categorized as: (1) infantile (birth to age 3 years), (2) juvenile (age 3 to 11 years), and (3) adolescent (11 years and older). 

These subclassifications are sometimes useful clinically, but have no established genetic basis. Age-specific genetic markers have not been identified for IS, and the current concept of scoliosis is that the disorder develops continuously between the juvenile years and adolescence. Hence, in this paper, the term IS is used in most situations without attempt to distinguish juvenile and adolescent subtypes. The incidence of IS for treatable curves defined as 25° or greater is greater in females than in males with a ratio of 2 : 1, respectively. Gender differences may underlie scoliotic curve progression.

Congenital scoliosis (CS) is a form of spinal curvature which is due to the presence of an underlying congenital vertebral malformation (CVM). 

**Figure 1:**
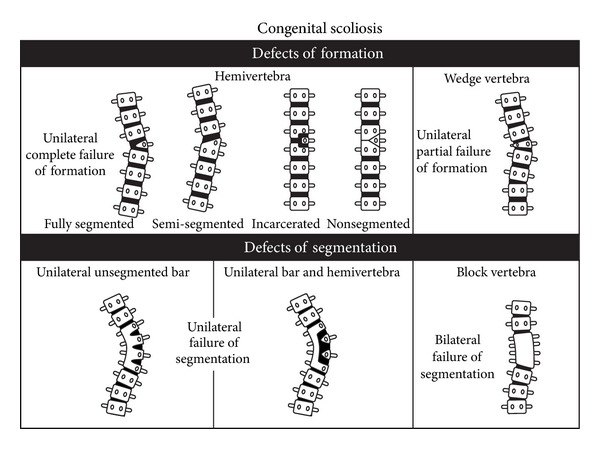
Diagram of spine illustrating defects of formation (wedge and hemivertebrae) and segmentation (vertebral bar and block vertebrae). Reprinted with permission from McMaster [6].

**Figure 2:**
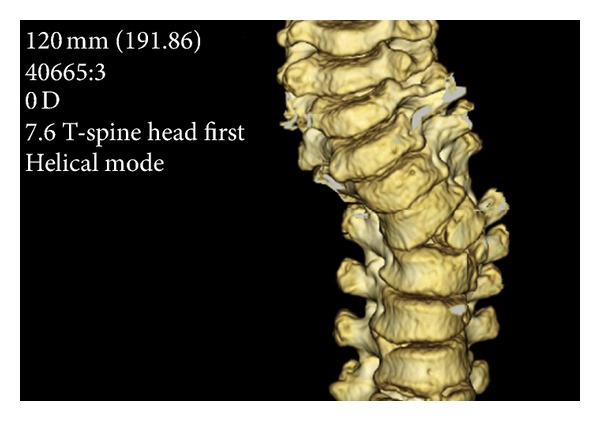
3D reconstruction illustrating congenital scoliosis. Left T4 hemivertebrae displayed. Courtesy of Dr. Kenneth Noonan.

The estimated frequency of CVM in the general population is in the range of 0.13–0.5/1,000 [4]. Vertebral malformations most commonly include hemivertebrae (half of a vertebrae), additional vertebrae, vertebral bar (an abnormality of vertebral separation during development), butterfly, and wedge-shaped vertebrae illustrated in Figures 1 and 2. Vertebral malformations may represent an isolated finding, occur in association with other renal, cardiac, or spinal cord malformations, or occur as part of an underlying syndrome or chromosomal abnormality. Autopsy of fetuses with anencephaly and myelomeningocele demonstrates the presence of cervical and thoracic CVM, suggesting a related etiology for both neural tube defects and CVM [5].

Frequently encountered syndromes associated with CVM include the following:

- Alagille syndrome (peripheral pulmonic stenosis, cholestasis, facial dysmorphism);
- Jarcho-Levin syndrome (short trunk dwarfism, multiple vertebral and rib defects with posterior rib fusion);
- Klippel-Feil syndrome (short neck, low posterior hairline, and fusion of cervical vertebrae), hemifacial microsomia (associated with craniofacial anomalies including microtia);
- Goldenhar syndrome (hemifacial microsomia and epibulbar dermoids); and
- VACTERL syndrome (vertebral malformations, anal atresia, cardiac malformations, tracheo-esophageal fistula, renal, and radial anomalies, and limb defects).

## 3. Vertebral Development and Genes Involved

Vertebral bodies are derived from somites through a recurrent process of budding off from the presomitic mesoderm mediated by cyclical expression of FGF, Wnt, and Notch signaling pathway genes [8]. A “clock and wavefront” model for somitogenesis was originally proposed by Cooke and Zeeman in 1976 [9]. In this model the “clock” represents an oscillator which connects presomitic mesodermal cells, and the “wave” represents a region of “rapid cellular change” in which transition to somite development occurs, presumably mediated by some type of gradient. 

A similar mechanism of oscillation amongst members of the Hes/Her/Hairy family of basic helix-loop-helix (bHLH) transcriptional repressors has been reported in mice, chicken, and zebrafish, providing evidence for conservation of the oscillator in vertebrates [10–13]. A molecular oscillator regulates the Notch, Fgf, and Wnt signaling pathways in which the Notch and Fgf genes oscillate in opposite phase to the Wnt genes [14]. Wnt3a signaling mediated by *β*-catenin which controls the oscillatory signaling in the Notch pathway [15]. Following periodic activation of Notch 1, Notch intracellular domain (NICD), the cleaved form of the Notch 1 receptor, translocates to the nucleus. NICD activates transcription of multiple target genes including Hairy/Hes/Her genes, Lunatic fringe (Lfng), and Notch-related ankyrn repeat protein (Nrap) [14, 16, 17].

**Figure 3:**
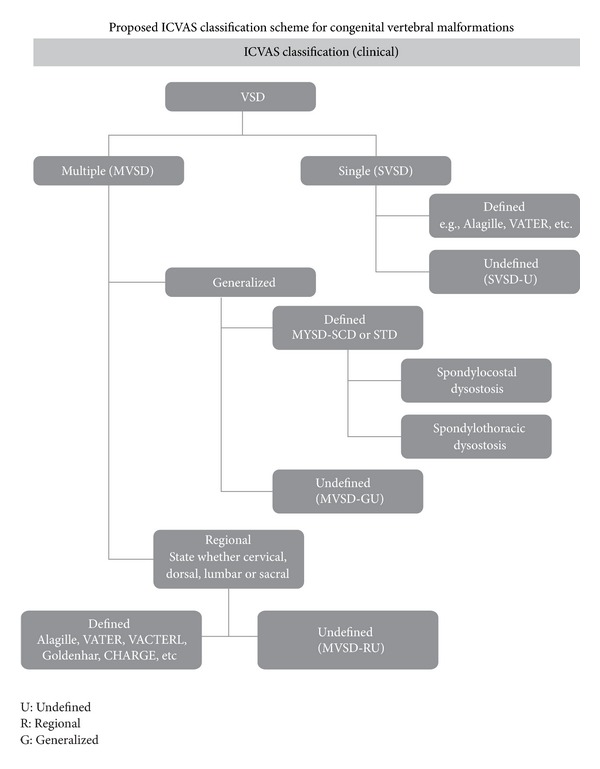
Algorithm for International Consortium for Vertebral Anomalies and Scoliosis (ICVAS) classification of congenital vertebral malformation. Reproduced with permission Expert opinion in [7]. Reproduced from Expert Opinion in *Expert Opin. Med. Diagn. (2008) 2(10):1107-1121* with permission of Informa UK Ltd.

A stripe of expression of genes occurs in response to the periodic clock signal at a region referred to as the determination front, which is defined by opposing retinoic acid (RA), FGF, and Wnt signaling gradients, posteriorly regressing as the embryo elongates along the anterior-posterior axis [15, 18, 19] Figure 3. The exposure of cells in the posterior presomitic mesoderm to high levels of FGF and Wnt activity enables the maintenance of an undifferentiated state [20, 21]. Below the determination front, cells are capable of responding to the segmentation clock through the activation of boundary specific genes Mesp2 and Riply [21–23]. Wnt3a provides a crucial function in both the clock and wavefront portions associated with somitogenesis and through Msgn1 plays a major role in the segmentation clock through regulation of Notch and Wnt signaling pathways [24]. As a result of active Wnt signaling, active Wnt signaling, Msgn1 and Wnt targets are expressed. A phase lag allows for Msgn1 to activate Notch related genes. RA plays an important role in the preservation of spine symmetry through its buffering action of the Left-Right pathway which creates asymmetry through the action of Nodal [25]. Since the majority of patients with IS exhibit a spinal curve to the right, an underlying defect in left-right asymmetry has been hypothesized [26].

## 4. Teratogens Associated with CVM

Various maternal exposures during pregnancy including alcohol use [27], anticonvulsant medications such as valproic acid [28–30], hyperthermia [31], maternal insulin-dependent diabetes mellitus, and gestational diabetes [32–34] have been observed to be associated with the occurrence of CVM in animal models and humans. Single nucleotide polymorphisms in glucose metabolizing genes including *GLUT1*, *HK1,* and *LEP *are postulated to be related to the occurrence of malformations observed in diabetic embryopathy. The occurrence of reactive oxygen species (ROS) has been proposed as a mechanism for altered somitogenesis in diabetic embryopathy [35]. Mutations in the planar cell polarity gene, *CELSR1* (*Caherin, EGF Lag Seven Pass G-Type Receptor 1–3)*, have been identified in patients with either neural tube defects or caudal agenesis [36]. Mutations in planar cell polarity genes are associated with a shortened body axis, widened neural plate, and neural tube defects [37]. CVM have been observed in laboratory animals exposed to I (Kr)-blockers (class III anti-arrhythmic agent), zinc deficient diet, the organophosphate pesticide chlopyrifos fumonisins (environmental toxins produced *Fusarium moniliforme* (*F. verticilliodes*), *F. proliferatum*, and other *Fusarium* species of molds), during pregnancy [38–40].

Fish with vertebral deformities and abnormal mechanical vertebral properties were produced following exposure of juvenile fourhorn sculpin, *Myoxocephalus quadricornis* L. to tetrachloro-1, 2-benzoquinone, a component in bleached kraft mill effluents [41]. Exposure to carbon monoxide[42] and boric acid are associated with alterations in HOX-mediated gene expression [43]. Retinoic acid, a vitamin A analogue, has been observed to cause homeotic transformations in mice and axial skeletal truncation in the *Dominant hemimelia (dh) *mouse, suggesting a possible relationship between retinoic acid signaling and the *dh* gene [44]. Increased axial skeletal defects and apoptosis were associated with inhibition of nitric oxide (NO) production or the addition of NO to developing chick embryos [45]. Low birth weight, decreases in successive births, and behavioral deficits replicated by carbon monoxide alone in animal models have been reported to occur in conjunction with cigarette smoking during pregnancy [46, 47]. Cigarette smoke generation of ROS resulting in somite anoxic damage could potentially contribute to the development of CVM. 

The occurrence of CS in monozygotic twins [48] is consistent with an observed increased risk for congenital malformations in both monozygotic and dizygotic twins [49]. Congenital malformations and syndromes including Prader-Willi, Angelman, and Beckwith-Wiedemann syndromes have been linked to assisted reproductive technology (ART) [50]. Methyl donor content of the growth media has been suggested as a possible mechanism of CVM occurrence in ART-assisted pregnancies, and nutritional factors have been implicated for their occurrence in non-ART pregnancies, suggesting a possible relationship between epigenetic factors and CVM. Similar to other birth defects, CVM often represent sporadic occurrences making epigenetic factors another plausible mechanism for investigation.

Hyperthermia has been associated with CVM development. Heat shock proteins are recruited when there is exposure to nonteratogenic doses of heat (<2°C) which provide protection for proteins against subsequent damage by teratogenic doses of heat (>2°C). Heat shock proteins attach to uncovered active sites, thus preventing their binding with other functionally impaired aggregate proteins [51]. Hyperthermia results in inhibition of the cell cycle and induces apoptosis. Although the exact mechanism responsible for altered somitogenesis associated with heat is uncertain, Notch/Delta signaling pathway proteins may undergo alteration(s) and result in abnormal vertebral patterning. 

Presently there are no reported studies which describe the relative contribution of maternal exposures to CVM development. In a series of 206, 244 live births, still births, and elective terminations, a total of 5 cases of isolated hemivertebrae, and 22 cases of hemivertebrae with other birth defects were identified [52]. The most common maternal exposure associated with CVM was maternal diabetes (5 cases) followed by twinning (2 cases).

## 5. Classification of CVM

**Figure 4:**
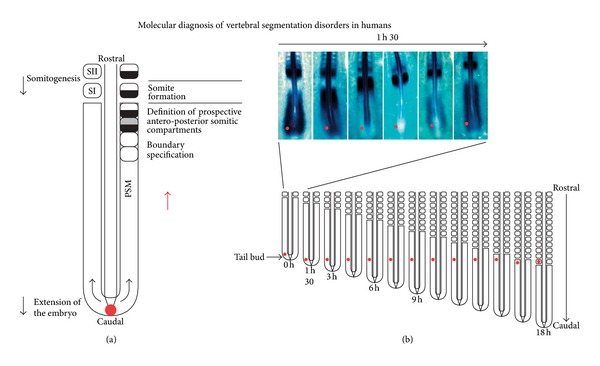
Illustration of somite formation from the presomitic mesoderm (PSM) in the chick embryo. Paired somites are formed every 90 minutes in a periodic fashion every 90 minutes shown in (a). (b).A molecular clocked linked to segmentation by dynamic and periodic expression of the cyclic genes in the PSM. Top: Lunatic Fringe mRNA expression appears as a wave sweeping across the whole PSM once during each somite formation as illustrated by in situ hybridization in this 17-somite-old chick embryo. During each somite formations, PSM cells illustrated by the dot undergo a phase of upregulation of the cycling genes followed by a phase of down regulation of these genes. Bottom: As shown in this schematic representation of the progression of somitogenesis in the embryo, the cycles of expression of the cyclic genes will last while the cells remain in the PSM, which corresponds approximately to the time to form 12 somites in the chick embryo. These PSM cells undergo 12 oscillations of the expression of the cycling genes. Reproduced with permission Expert Opinion in [9]. Reproduced from Expert Opinion in *Expert Opin. Med. Diagn. (2008) 2(10):1107-1121* with permission of Informa UK Ltd.

A classification scheme for CVM which is simple and unified is essential for clinicians and researchers to describe individual and collective CVM from both a phenotypic and genetic etiologic vantage point. A number of classification schemes for CVM have been proposed which have individually focused upon different components associated with CVM, an occurrence including a developmental basis for CVM, [53, 54] syndromic diagnosis of CVM (i.e. spondylocostal dysostosis, Klippel-Feil, etc.) [54–56], and mode of inheritance [57]. Recently a proposed pilot classification system by the International Consortium for Vertebral Anomalies (CVM) and Scoliosis (ICVAS) was outlined algorithmically in Figure 4 [58]. A category of vertebral segmentation defects (VSDs) may be defined as a single (SVSD) or multiple (MVSD). Known syndromes such as hemifacial microsomia or VACTERL may be associated with an SVSD. MVSDs are defined as generalized when there is involvement of 10 or greater contiguous vertebral bodies and may represent a defined phenotype such as spondylocostal dysostosis or spondylothoracic dysostosis, or an undefined phenotype. Alternatively, MSVD may have a regional distribution and be associated with a defined or undefined phenotype. Since prior usage of the term “Jarcho Levin syndrome” has been associated with a wide range of inconsistent skeletal features, and has been used indiscriminately, ICVAS has recommended that this term not be used. A high degree of inter observer reliability has been noted with the proposed classification system, which provides a basis for future cohort genetic analysis of similar CVM phenotypes.

## 6. Monogenic CVM

**Figure 5:**
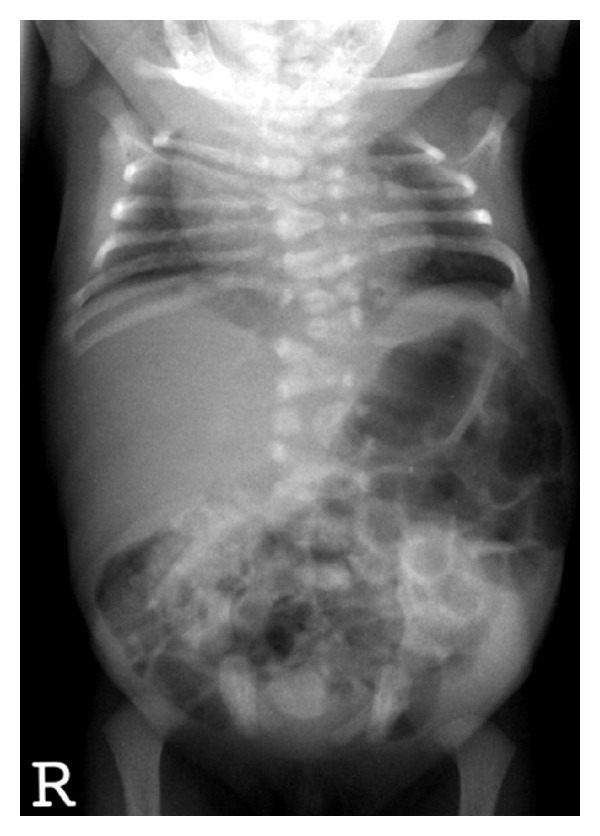
Radiographic features of spondylocostal dystostosis including contiguous vertebral malformations with asymmetric rib malformations. Photograph courtesy of Peter D. Turnpenny M.D., Royal Devon and Exeter Hospital.

Mutations in Notch signaling genes have been identified in two monogenic forms of CVM. Spondylocostal dysostosis (SCD) is an autosomal recessive disorder, with occasional autosomal dominant inheritance. Radiographically, SCD is characterized by contiguous vertebral segmentation defects in addition to rib abnormalities Figure 5. Affected individuals have disproportionate short stature, characterized by a shortened trunk and protuberant abdomen. Associated features include scoliosis and mild respiratory compromise. Mutations in *DLL3*, a Notch pathway signaling gene, were identified in Arab-Israeli and Pakistani kindreds using synteny conversion analysis [59]. Mutations in Notch signaling pathway genes, including *MESP2* [60],* LFNG* [61], and *HES7,* have subsequently been identified [62]. The term “pebble beach” sign refers to morphologically abnormal vertebral bodies characterized by a smooth, round contour, usually associated with the presence of a *DLL3* mutation [63]. Hypoplasia of the left vertebral artery has been reported in one affected individual with a compound heterozygous mutation in *HES7* (158D/V186Y).

**Figure 6:**
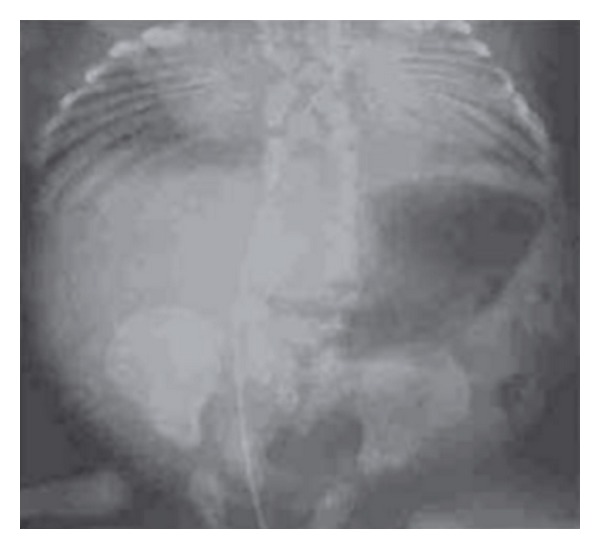
Radiograph features of spondylothoracic dysostosis demonstrating contiguous vertebral malformations with symmetric posterior rib fusion. Reproduced with permission Expert Opinion in [9].

Spondylothoracic dysostosis (STD) is an autosomal recessive disorder of vertebral segmentation with a clinical phenotype of disproportionate short stature, with increased thoracic anterior posterior diameter. STD has a radiographic appearance characterized by the presence of posterior rib fusion, also referred to as a “crab like thorax,” as illustrated in Figure 6 [64]. There is some degree of respiratory compromise due to the presence of the short thoracic cage. STD is caused by mutations in the MESP2 gene, and has a prevalence of 1/12,000 in the Puerto Rican population, with a suggestion of a founder effect of the E103X (p.Glu103X) mutation among Puerto Ricans [65]. Only 25% of affected children with STD survive into adolescence and adulthood, indicating that the degree of respiratory compromise is more severe in STD as compared to SCD. Thoracic insufficiency syndrome is associated with STD and is associated with underlying diminished lung volume and chest wall stiffness. CVM can be associated with a variety of syndromes as shown in Table 1.

## 7. Sporadically Occurring CVMs

Because CVM and associated syndromes usually represent sporadic occurrences, even within a particular family, it is difficult to identify causal genetic factors. A panel of genes associated with vertebral patterning defects including *PAX1, DLL3, SLC35A3, WNT3A, TBX6, *and* T (Brachyury) *were sequenced by our group in 50 patients with heterogeneous types of CVMs [66–70]. A mutation (c.1013C>T) resulting in an alanine to valine change was found at amino acid position 338 in the *T (Brachyury)* gene in three affected patients, in this cohort that was not present among 886 chromosomes in the CEPH diversity panel [66]. Collectively these patients had maternal pregnancy exposure histories of diabetes, valproic acid, and clomiphene. The third affected individual did not have any history of maternal exposure during pregnancy. The phenotypes of these patients were all distinct and included cervical and thoracic CVM and sacral agenesis. This mutation had previously been described in another individual with sacral agenesis with no history of maternal diabetes during pregnancy [71]. Although no mutations in *TBX6* were identified in the previously described patient series, polymorphisms of the somite patterning gene *TBX6*, specifically rs2289292 (located at exon 8) and rs380962 (located at the 5′UTR), may have an important role in the pathogenesis of congenital scoliosis in the Chinese Han population [72].

CVM may mediated through complex interactions of genetic, environmental and epigenetic factors. Gestational hypoxia in Hes7^+/-^ and Mesp2^+/-^ mice results in an increase in severity of CVM in mice. This effect mediated by abnormal FGF signaling results in altered somitogenesis and provides evidence that an environmental trigger such as hypoxia can potentiate a CVM occurrence in a genetically susceptible background [73]. The observation that the phenotypic expression of tail kinks in the axin fused mouse (*Axin* ^*Fu*^) can be altered by increased DNA methylation supports an epigenetic contribution to CVM occurrence [74].

Whole exome sequence (WES) and whole genome sequence (WGS) platforms represent suitable platforms for the identification of candidate gene sequence variants and copy number variants (CNV). WES analyzes approximately 1% of the entire genome and highlights identification of sequence variation in the coding and splice site regions in annotated genes identifying approximately 20,000 sequence variants. WGS is capable of uncovering all genetic and genomic variations, including single nucleotide variants (SNV) and CNV identifying approximately 3.5 million sequence variants [75]. A variety of filtering algorithms, including elimination of sequence variants, present in databases such as dbSNP and the 1,000 Genomes Project database, are implemented to narrow down potential candidate genes. Among coding variants decreasing priority is given to nonsense, frameshift, splice-site, and missense mutations. Inheritance modeling (dominant, recessive) computer prediction in conjunction with disease specific information helps to enable further refinement.

Evidence for localization of vertebral patterning genes identified in mice, Xenopus, and chickens, in synteny blocks supports a hypothesis for conservation of vertebral patterning genes among amniotes [76]. SNV identified in patterning genes previously identified in model organisms should be sought initially, although the advantage of WES and WGS is the ability to identify novel genes and pathways associated with disease. Following identification of a narrowed and focused list of candidate genes, functional confirmation is necessary. WES is applicable for the identification of SNV in highly penetrant mendelian disease phenotypes, whereas WGS has applications for both mendelian and complex phenotype identification in addition to sporadic phenotypes which are the result of *de novo* CNVs or SNVs.

## 8. “Sporadically” Occurring CVM-Related Syndromes

Oculo-auriculo-vertebral spectrum disorders and Klippel-Feil syndrome are two frequently encountered syndromes associated with CVM. Progress has been made in understanding their etiologies and each is discussed below.

### 8.1. Oculo-Auriculo-Vertebral Spectrum (Hemifacial Microsomia)

Major clinical features of oculo-auriculo-vertebral spectrum (OAVS) include unilateral microtia, craniofacial asymmetry, mandibular hypoplasia, ocular epibulbar dermoid, and CVM [77]. Additional features include: cleft lip with or without cleft palate, congenital heart disease, and congenital renal malformations. There is overlap between OAVS and other syndromes including Treacher Collins syndrome (associated with microtia, lower eyelid colobomas, and mandibular hypoplasia), Fanconi Anemia (radial ray abnormalities, short stature, elevated diepoxy butane induced chromosome breakage), and VACTERL syndrome. At the present time there is no common etiology for OAVS, although there is evidence supporting vascular disruption [78], maternal diabetes [79], and other teratogenetic agents including retinoic acid [80] and thalidomide [81]. Using high density oligonucletotide microarray CGH technology, 12 of 86 (14%) patients with hemifacial microsomia studied were identified as having a CNV, including 4 patients with deletions and/or 8 patients with duplications ranging between 2.3–2.8 Mb in size [82]. Of the three patients with CVM who had CNV, one patient had a paternally inherited 9q34.11 duplication. None of the genes involved in the 9q34.11 have any known function with respect to vertebral body development; a second patient had a duplication involving 20p12.2. The *ANKRD5* gene was present within this region and is not known to have any known function in somite formation; the third patient had a coincident isodicentric Y chromosome. These results indicate that CNV represents a minority of genetic causes for hemifacial microsomia and support a hypothesis for genetic heterogeneity of OAVS.

### 8.2. Klippel Feil Syndrome

The majority of cases of Klippel-Feil syndrome (short neck, low posterior hairline, and fusion of cervical vertebrae) represent sporadic occurrences within a family. However, Klippel-Feil syndrome may represent a familial occurrence in which multiple family members are affected. Autosomal dominant, autosomal recessive, and X-linked forms of Klippel-Feil syndrome have been reported [83]. Wildervank syndrome refers to a constellation of features including Klippel-Feil syndrome, congenital hearing loss, Duane retraction syndrome (limitation of abduction with narrowing of the palpebral fissure and retraction of the globe) [84].

Klippel-Feil syndrome is sometimes associated with mirror movements, or the involuntary movement of the one extremity mimicking the opposite extremity, with a central mirror serving as a reference point, reflecting the image of the voluntary extremity to the opposite side [85–88]. One neuroanatomic basis for mirror movements is hypothesized to be related to variations in the normal pathways of descending corticospinal tracts, including the crossed lateral corticospinal tract (LCT), uncrossed anterior corticospinal tract (ACT), and anterolateral corticospinal tract (ALCT) [88]. Other hypotheses include delayed resolution following a CNS insult or loss of normal control pathways. No coding mutations were identified in a series of genes associated with aberrant ocular motor and corticospinal axon path development in a patient with Wildervanck syndrome, mirror movements and neuroschisis, including *ROBO3*, *CHN1*, *HOXA1*, *DCC,* and *GDF6* [89]. Analysis of additional patients would be helpful to support a hypothesis for mutations in genes associated with corticospinal axon path development.

A mutation at a highly conserved region in the BMP ligand *GDF6* gene c.866T>C was identified in both familial and sporadic forms of Klippel Feil syndrome [90]. The variable expressivity in affected family members and incomplete penetrance observed in *GDF6* knockout mice suggest thresholds of GDF6 necessary for spine development are subject to modification by environmental factors and may vary between individuals and within different spinal regions. An autosomal dominant mutation (R266C) in *GDF3* has been identified in one family with ocular defects including iris and retinal coloboma and CVM [91]. Zebrafish morpholinos for *Gdf1/3* demonstrated retinal colobomas and trunk shortening with vertebral malformations. 

## 9. Idiopathic Scoliosis

### 9.1. Management

While CS is associated with underlying CVM, the spine in IS has a normal morphologic appearance. The incidence of IS for treatable curves defined as 25° or greater is greater in females than in males with a ratio of 2 : 1, respectively. Gender differences may underlie scoliotic curve progression [92].

Current management of IS in a growing child includes: (1) Observation of curves that are <25°, (2) Bracing fulltime for curves progressing >25°, and (3) surgery (spinal fusion and instrumentation) for curves >40–45°. By age 16, 0.6% of affected people will have required active treatment with a full-time thoracolumbar-sacral orthosis (TLSO) or surgical correction with instrumentation [3]. Bracing involves the wearing of a TLSO 22 hours/day until spinal maturation [93]. Fulltime bracing is 80%–85% effective in holding curves under the surgery range at the completion of growth. However, in spite of full compliance with brace wear, there is a 15%–20% failure of bracing, and surgery is indicated.

Though scoliosis manifests during adolescence, it continues to cause significant medical problems most of late adolescent and adult life. The population of scoliotic teenagers treated in the 1950s and 1960s has now reached adulthood. Those who underwent surgical corrections are now manifesting the late effects of both the underlying scoliosis and interventional outcomes. Those who had no surgical intervention also manifest the later effects of scoliosis: back pain, progression, and significant respiratory and cardiac compromise [94]. These late consequences are not surprising in light of the pathological consequences associated with the disorder. Significant health problems have been reported in association with IS, including chronic back and neck pain, flatback syndrome, disc herniations, osteoarthritis, osteoporosis, kyphosis, disability, cosmetic dissatisfaction, and psychologic distress [95]. Patients with severe scoliosis, that is, curves >70°, are 3 times more likely to die from cardiopulmonary disease than unaffected individuals [96].

### 9.2. Genetic Etiologies of IS

The mode of inheritance of IS has not been solidly established and is under debate [1, 26, 95, 97–100]. Inheritance patterns reported include autosomal dominant with variable penetrance, autosomal recessive, multifactorial, and X-linked dominant modes. Hypotheses advanced to explain pathogenesis of IS include abnormalities in the composition of the connective tissue matrix, melatonin, calmodulin, neuromuscular imbalance, and altered vestibular function. Previous studies, illustrated in Table 2, demonstrated genetic heterogeneity for IS, although no single gene linked with the development of IS has been identified to date.

Candidate gene analysis of IS has focused on stratification of genes on the basis of their presumed function including: connective tissue, bone formation and metabolism, melatonin signaling pathway, puberty, and growth [115]. Several genes encoding extracellular matrix proteins, including elastin, types I and II collagen (COL1A1, COL1A2, COL2A1), and fibrillin, failed to demonstrate linkage to IS [97, 116]. Melatonin is considered a contributor to IS based on the observation that pinealectomy in newborn chickens leads to a spinal deformity similar to IS in humans [117]. Melatonin signaling was also impaired in patients with IS [118]. However, no evidence for linkage of IS to chromosome 4q, the locus for the human melatonin 1A receptor, has been observed, indicating that scoliosis does not result solely from melatonin deficiency [105, 119].

Linkage to 19p13 was described in two separate studies [102, 108]. Two loci within this region are credible candidates for IS: fibrillin 3 and thromboxane A2 receptor. Fibrillin 3 is a component of the extracellular matrix, which contributes to microfibrillar structure. Since abnormalities in platelet function have been reported in IS [120, 121], attention has turned towards understanding the interaction between calmodulin, myosin, and actin in platelets and subsequent development of IS.

These studies described above were largely based on analysis of strategically spaced genetic markers across the genome in large families with IS in order to identify linkages to a chromosomal region corresponding to the potential genetic basis for IS. Further exploration of candidate gene region(s) demonstrating association with familial IS would be required to determine their relative contribution to isolated sporadic (non familial) cases of IS.

### 9.3. Genetic Prognostic Factors Associated with IS and Curve Progression

Why and which curves will fail treatment are not known. Theories abound as to hypokyphotic curves, larger magnitude curves, and less flexible curves. There is evidence that genetic factors such as estrogen receptor genotype may predict curve progression in IS [122]. There is also evidence that elevated calmodulin levels contribute to curve progression in IS, possibly through interference with estrogen binding to the estrogen receptor [123]. SNPs have been used to develop an AIS-Prognostic Test (AIS-PT) to identify the curves that will not require bracing or surgery and curves that will fail bracing.

Determining which children with adolescent idiopathic scoliosis (AIS) between the ages of 9 and 13 years will require bracing is a challenge for the treating orthopedic surgeon. An application of genetic knowledge is to use this information in combination with additional clinical information to determine which patients using a series of 52 single nucleotide polymorphisms associated with genetic loci on all chromosomes except 3, 13, 21, and the Y chromosome, in conjunction with a the Cobb angle at the time of initial diagnosis, a logistic regression analysis has been utilized to obtain an AIS Prognostic Test score between 1 and 200 [124]. In three tested populations, low risk scores of <41 were observed to have a negative predictive value of 100%, 99%, and 97%. High risk scores (181–200) would identify the 1-2% of patients most likely to progress to a severe curve. Those patients with intermediate risk scores (51–180) would require close follow up for their curve progression by an orthopedic surgeon. Presently, information regarding the biological function of the genes used for the AIS Prognostic Test score is incomplete. The potential advantage of prognostic testing would be to reduce costs of imaging in those patients who are at a lower risk for scoliosis curve progression.

In addition to previous studies suggesting a genetic component linked to the development of IS as a binary trait, there is evidence that genetic factors may predict curve progression in IS. An association study performed in 304 females with IS demonstrated a significantly greater Cobb angle at the time of growth maturation among patients with estrogen receptor genotype XX and Xx compared to patients with genotype xx (*P* = 0.002) [122]. A higher risk for operative treatment was observed among patients with genotype XX and Xx, compared to patients with genotype xx. There is also evidence that elevated calmodulin levels contribute to curve progression in IS, possibly through interference with estrogen binding to the estrogen receptor [123]. 

The single-nucleotide polymorphism SNP-418G/C in the tissue inhibitor of metalloproteinase-2 gene promoter region was associated with thoracic scoliosis curve severity [125]. Downregulation of *TIMP-2* transcriptional activity resulting in increased vascular proliferation and enhanced anterior spine endochondral ossification during adolescence could result in disproportionate spinal growth and result in thoracic scoliosis. The promoter polymorphism (rs11063714) in the neurotrophin 3 (*NTF3*) gene is associated with curve severity for IS in the Chinese Han population. Individuals affected with IS having an AA genotype had lower mean maximum Cobb angle as compared to patients with AG and GG genotypes [126]. Patients who were skeletally mature and had an AA genotype had greater success for treatment with bracing as compared to patients with GG genotype. Egr 3^−/−^ mice fail to express NTF3 and have proprioceptive dysfunction due to muscle spindle agenesis, apoptosis of proprioceptive neurons, proprioceptive neuron apoptosis, and disruption of synaptic connectivity between muscle sensory and motor neurons. A reduction in the number of muscle spindles and malfunction has been demonstrated in spinal muscle obtained from patients with IS, examined histologically and histochemically [127]. There is also increased expression of NTF3 messenger RNA in paravertebral muscle in IS [119]. These observations in addition to a strong linkage signal on chromosome 12p13 [111], the *NTF3* locus provide support for a role of *NTF3* in the pathogenesis of IS.

The above summary illustrates the difficulty of identifying causative genes for IS lies in extreme phenotypic and genetic heterogeneity. Future research will need to be aimed at improved stratification of clinical cases based on factors such as age of onset, curve progression, severity, responsiveness to bracing, and correlation with mutations in genes identified using next generation sequence platforms such as whole exome and whole genome analysis [115].

## 10. Relationship between Congenital and Idiopathic Scoliosis

Multiple studies support a common genetic etiology for congenital and idiopathic scoliosis. A family history of IS was observed in 17.3% of 237 families in which an affected proband had congenital scoliosis [128]. In 52 families with IS a significant linkage peak was observed on chromosome 8q12 (multipoint LOD 2.77; *P* = 0028). Over transmission of the CHD7 associated polymorphism, *rs4738824* in patients with IS was observed in a cohort of 52 families. Substitution of the A allele of this polymorphism with the G allele is predicted to disrupt a possible binding site for caudal-type (cdx) homeodomain-containing transcription factors. Mutations in *CHD7*, a chromeodomain helicase DNA binding protein are associated with CHARGE syndrome (coloboma of the eye, heart defects, atresia of the choanae, retardation of growth and/or development, genital and/or urinary abnormalities, and ear abnormalities and deafness) [129]. A hypothesis for the development of idiopathic scoliosis is CHD7 may act postnatally to alter spinal growth during the adolescent growth spurt. Chd7 in zebrafish is expressed in somites, brain, eye, and otic vesicle. *Chd7* enables proper symmetric expression of critically important somitogenesis associated genes located downstream from Wnt including* her7*, *cdx1a*, *dlc*, *mespa*, and *ripply*. Zebrafish morpholinos in which CHD7 was knocked down were noted to have tail kinks and a progressively shortened axis [130]. Chd7 plays an important role in somitogenesis as supported by a lack of distinct somite boundary formation and abnormal expression of *ephrin B2a*, an important segment polarity gene when this gene is knocked down in zebrafish [131]. Knockdown of lysyl oxidases lox11 or lox15b in zebrafish in conjunction with diminished copper availability result in distortion of the notochord formation, suggesting a relationship between genetic and nutritional factors in the etiology of notochord development. However no association between coding or tag SNPs in *LOX, LOXL1, LOXL2, LOXL3, LOXL4,* and idiopathic scoliosis was observed.

## 11. Summary

While CS and IS represent clinically distinct conditions, there is evidence supporting a hypothesis for a common pathogenetic mechanism. The underlying genetic etiologies and respective environmental contributions have not been delineated. The obstacles which need to be overcome include clinical heterogeneity with respect to diversity of the types of CVM with contribute to CS. Idiopathic scoliosis is also a clinically heterogeneous condition and is associated with different ages of onset and prognoses. Advances in genetic technologies can assist in the identification of sequence variants which may contribute to the occurrence of both conditions. Challenges for both conditions are to evaluate their relative contribution to the development of CVM or IS, in addition to determine how multiple mutations in a single individual may interact with one another and environmental factors. The treatment of both conditions requires a multispecialty approach. Unraveling the genetic contributions for both conditions can help to provide improved genetic counseling, prevention, and treatment strategies for families.

## Figures and Tables

**Table 1:** Some syndromes that include congenital vertebral malformations.

Syndrome	OMIM reference	Corresponding gene(s)
Acrofacial dysostosis*	263750	
Aicardi*	304050	
Alagille	118450	*JAGGED1, NOTCH2 *
Anhalt*	601344	
Atelosteogenesis III	108721	*FLNB *
Campomelic dysplasia	114290	*SOX9 *
Casamassima-Morton-Nance*	271520	
Caudal regression*	182940	
Cerebro-facio-thoracic dysplasia*	213980	
CHARGE	214800	*CHD7 *
“Chromosomal”		
Currarino	176450	*HLXB9 *
DeLa Chapelle*	256050	
DeGeorge/Sedlackova	188400	Microdeletion, 10p13-p14, 22q11.2,
Dysspondylochondromatosis*		
Femoral hypoplasia-unusual facies*	134780	
Fibrodysplasia ossificans progressive	135100	*ACVR1 *
Fryns-Moerman*		
Goldenhar*(Oculo-auriculo-vertebral spectrum)	164210	
Incontinentia Pigmenti	308300	*NEMO *
Kabuki	147920	*MLL2 *
Kaufman-McKusick	236700	*MKKS *
KBG Syndrome*	148050	
Klippel-Feil*	118100	?*PAXl, GDF6 *
Larsen	150250	*FLNB *
Lower mesodermal agenesis*		
Maternal diabetes*		
MURCS Association*	601076	
Multiple Pterygium Syndrome	265000	*CHRNG *
OEIS Syndrome*	258040	
Phaver*	261575	
Rapadilino	266280	*RECQL4 *
Robinow	268310	*ROR2 *
Rolland-Desbuquois*	224400	
Rokitansky Sequence*	277000	?*WNT4 *
Silverman	224410	*HSPG2 *
Simpson-Golabi-Behmel	312870	*GPC3 *
Sirenomelia*	182940	
Spondylocarpotarsal Synostosis	272460	*FLNB *
Spondylocostal Dysostosis	277300	*DLL3, MESP2, LFNG *
Spondylothoracic Dysotosis*	277300	*MESP2 *
Thakker-Donnai*	227255	
Toriello*		
Urioste*		
VATER/VACTERL*	192350	
Verloove-Vanhorick*	215850	
Wildevanck*	314600	
Zimmer*	273395	

*Underlying cause not known. Reproduced from *Expert Opinion in Expert Opin. Med. Diagn. (2008) 2(10):1107-1121* with permission of Informa UK Ltd.

**Table 2:** Summary of prior genetic linkage studies for IS.

Study	No. of Families/Individuals	Region(s)	Model	Comments
Wise et al. [101]	1/14	6q distal 10q 18q	Autosomal dominant	Genome wide search in one family of French Acadian and English descent (7 affected members), with validation of “hot spots” in a second large family

Chan et al. [102]	7/52	19p13.3	Autosomal dominant	Recruited Asian patients in whom scoliosis developed in adolescence

Baghernajad Salehi et al. [103]	1/17	17p.11	Autosomal dominant	3 generation Italian family

Justice et al. [104]	202/1198	Xq23 Xq26.1	X-linked dominant	Maximum lod score of 1.69 (theta = 0.2) identified at marker GATA172D05. A lod score of 2.23 for this marker was found in one family with six affected individuals

Morcuende et al. [105]	47/176	4q35	N/A	No linkage to *MTNR1A* (Melatonin Receptor 1A) and no mutations in *MTNR1A *

Bashiardes et al. [106]	7 individuals	8p23.2-8q11.21	Autosomal dominant	Pericentric inversion in chromosome 8 disrupts *SNTG1* (syntrophin). Five of 7 individuals in family have *SNTG1 *deletion

Miller et al. [107]	202/1198	6, 9, 16 and 17	Autosomal dominant	Model independent linkage analysis

Alden et al. [108]	202/1198	19p11.3	Autosomal dominant	Threshold of curvature set at 30°. Fibrillin 3, thromboxane A2 receptor, possible candidates

Baghernajad Salehi et al. [103]	1500 individuals	Chromosome 3 Chromosome 7	Autosomal dominant	Patients' familial relationships established through database

Gao et al. [109]	52	8q	N/A	CHD7 Gene polymorphisms are associated with susceptibility to idiopathic scoliosis

Ocaka et al. [110]	25/208	9q31.2-q34.2; 17q25.3-qter	Autosomal dominant	Confirmation of 9q [107]

Raggio et al. [111]	7/48	12p13.3	Autosomal dominant; autosomal recessive	All families contribute to recessive model. 5/7 families contribute to the dominant model

Gurnett et al. [112]	1/22	18q	Autosomal dominant	LOD score 3.86 Scoliosis and pectus excavatum

Sharma et al. [113]	419	3p26.3 (*P* < 8 × 10^−8^)	N/A	GWAS study. *CHL1*, *DSCAM, CNTNAP2 *genes involved in axon guidance

Takahashi et al. [114]	1050	*LBX1* (*P* = 1.24 × 10^−19^)	N/A	GWAS study. LBX1 is determinant of dorsal spinal neurons; altered somatosensory function
